# Confirmed record of *Boulenophrys binchuanensis* (Anura, Megophryidae) from Jiulong County, Sichuan Province, China, with notes on tadpoles and advertisement calls

**DOI:** 10.3897/BDJ.13.e165212

**Published:** 2025-08-21

**Authors:** Tianyu Qian, Wenhui Liu, Huaming Zhou, Yujuan Guo, Feirong Ji, Cheng Li, Jianping Jiang

**Affiliations:** 1 Chengdu Institute of Biology, Chinese Academy of Sciences, Chengdu, China Chengdu Institute of Biology, Chinese Academy of Sciences Chengdu China; 2 Ganzi Institute of Forestry Research, Kangding, China Ganzi Institute of Forestry Research Kangding China; 3 Mangkang Biodiversity and Ecological Station, Xizang Ecological Safety Monitor Network, Changdu, China Mangkang Biodiversity and Ecological Station, Xizang Ecological Safety Monitor Network Changdu China

**Keywords:** biodiversity monitoring, ecology notes, frogs, natural history

## Abstract

**Background:**

The horned frog population, previously identified as *Boulenophrys minor* from Jiulong County, Sichuan Province, China, was recently proposed to be *B. binchuanensis*, based on geographical inference, which remains unconfirmed by specimen examination.

**New information:**

We revisited this population and confirmed its identification as *Boulenophrys binchuanensis* in Jiulong County using molecular data from 16S and CO1 genes, complemented by morphological diagnosis. Additionally, we described its tadpoles and advertisement calls, extending the known range and informing conservation efforts.

## Introduction

The Binchuan horned frog, *Boulenophrys binchuanensis* (Ye and Fei, 1995), was originally described as a subspecies of *B. minor* (Stejneger, 1926), based on a small population in Yunnan Province, China. The original description noted “intermediate populations” between the type localities of *B. binchuanensis* and *B. minor*, which were assigned to *B. minor* due to morphological similarities (Ye and Fei 1995). Recent molecular studies have clarified that *B. minor* has a narrow distribution, restricted to Dujiangyan, Sichuan Province (Chen et al. 2017, Liu et al. 2018). Sebsequently, Lyu et al. (2023) re-examined the "intermediate population" from Yanbian County, Panzhihua, Sichuan Province and revised it as *B. binchuanensis*. Based on biogeographical inference, Lyu et al. (2023) also tentatively revised other “intermediate populations” in Wuding and Qiaojia (Yunnan Province) and Huili, Jiulong and Muli (Sichuan Province) as *B. binchuanensis*, though these lack genetic confirmation.

During field surveys in 2023, we investigated the *Boulenophrys* population in Jiulong County, Sichuan Province. We collected molecular samples from adults and tadpoles and recorded bioacoustics data. Here, we confirm the presence of *B. binchuanensis* in Jiulong County and provide notes on its tadpole morphology and advertisement calls.

## Materials and methods

Three adult specimens were collected on 6 August 2023 and four adult specimens and twelve tadpole specimens were collected on 23 August 2023 from Xiaojinxiang, Jiulong County, Sichuan Province, China (28.662°N, 101.925°E, ca. 2540 m a.s.l.). Specimens collected during the first survey were directly fixed with 70% alcohol and those collected during the second survey were fixed with 10% formalin for several days and then transferred to 70% alcohol for storage. Tissue samples were extracted prior to fixing and preserved in 95% ethanol. All samples were deposited in the Chengdu Institute of Biology (CIB), Chinese Academy of Sciences (CAS).

For molecular analysis, we extracted segments of the 16S ribosomal RNA gene (16S) from all newly-collected specimens using primer pair 16Sar-L (5′-CGCCTGTTTACCAAAAACAT-3′) and 16Sbr-H (5′-CGCCTGTTTACCAAAAACAT-3′) from Palumbi et al. (1991) and cytochrome C oxidase subunit I gene (CO1) using Chmf4 (5′-TYTCWACWAAYCAYAAAGAYATCGG-3′) and Chmr4 (5′-ACYTCRGGRTGRCCRAARAATCA-3′) from Che et al. (2012). The amplification protocol was performed following Ji et al. (2023). Sequencing was conducted using an ABI3730 automated DNA sequencer from Chengdu Youkang Jianxing Biotechnology Co., Ltd. (Chengdu, China). We obtained 16S segments from 19 specimens (GenBank Accession Numbers: PV826423–441) and CO1 segments from 16 specimens (GenBank Accession Numbers: PV826486–501). The new sequences were then searched on BLAST (NCBI) to verify their approximate identity. After that, we downloaded all sequences of *B. binchuanensis* reported by Chen et al. (2017) and Lyu et al. (2023) from GenBank, including three samples from Mt. Jizu, Yunnan Province (KIZ 019441 [GenBank Accession Number for 16S: KX811849; for CO1: KX812112], SYS a007843 [OQ180976, OQ180864], KIZ 019442 [KZ811850, KX812113]), one sample from Mt. Huafo, Yunnan Province (KIZ 011261 [KX811848, KX812111]), one sample from Mt. Ailao, Yunnan Province (KIZ YPX32023 [KX811851, KX812114]) and one sample from Yanbian, Sichuan Province (SYS a006821 [OQ180970, OQ180858]). We then aligned the new sequences of the 16S and CO1 genes with the downloaded sequences, respectively, using the MUSCLE algorithm (Edgar 2004) with default parameters and calculated the uncorrected *p*-distance of the 16S gene using MEGA 12 (Kumar et al. 2024). For reconstructing the phylogeny, we combined the new alignment with the dataset reported by Qian et al. (2023c), which contains 545 bp for 16S and 629 bp for CO1. The phylogenetic tree was constructed using Bayesian Inference (BI) implemented in MrBayes 3.2.7a (Ronquist et al. 2012). Two independent runs were conducted during the BI analyses with 10,000,000 generations each, sampled every 1000 generations, with the first 25% of samples discarded as burn-in, resulting in a potential scale reduction factor (PSRF) of < 0.005.

Morphological measurements of adult specimens were taken by a digital caliper, definitions followed Lyu et al. (2021) and were modified by Qian et al. (2023c): snout-vent length (SVL) from tip of snout to vent; head length (HL) from the point behind the angle of the jaw to tip of snout; head width (HW) at the widest point; snout length (SL) from the tip of snout to the anterior corner of the eye; internasal distance (IND); interorbital distance (IOD), the minimum distance between upper eyelids; eye diameter (ED); tympanum diameter (TD); tympanum-eye distance (TED) from the anterior edge of tympanum to posterior corner of the eye; hand length (HNL) from the proximal border of the outer palmar tubercle to the tip of finger III; length of lower arm and hand (LAHL), from the flexed elbow to the tip of finger III; tibia length (TL) from the outer surface of the flexed knee to the heel; foot-tarsus (FTL) length from the distal end of the shank to the tip of digit IV; and foot length (FL) from the tip of third digit to the base of inner metatarsal tubercle. Sex was determined by the calling behaviour of males, the presence of nuptial pads in males and/or gonadal inspection.

Tadpoles were measured using Image J 1.54g software (Schneider et al. 2012) from photographs of preserved specimens taken next to a scale. Definitions followed those of Oberhummer et al. (2014): body height (BH), maximal body height at trunk; body length (BL) from snout to the point where the axis of the tail myotomes meets the body wall; body end to the centre of spiracle (BS); maximal body width (BW); eye diameter (ED); eye-snout distance (ESD); the internarial distance (IND) measured from centre to centre; the interorbital distance (IOD) measured from centre to centre; maximal tail height (MTH); lower fin height (LFH) at MTH; upper fin height (UFH) at MTH; distance from the centre of naris to the centre of the eye (NED); oral disc width (ODW); distance from snout to the centre of naris (SND); distance from snout to the centre of spiracle (SSD); total length (TTL); tail length (TAL) = TTL – BL; tail muscle height (TMH) at the body-tail junction, where ventral line of musculature meets trunk contour; and tail muscle width (TMW) at the same level as TMH. The ODW was measured in expanded form by anchoring it to a glass following Qian et al. (2023b). Tadpole staging followed Gosner (1960) and the descriptive terminology for external morphology followed Altig and McDiarmid (1999).

We made recordings of advertisement calls (CIB RC230084, 081 and 083) from three males (CIB SCJL20230823001, CIB SCJL20230823003 and CIB SCJL20230823004) during the second survey using a Zoom F3 digital recorder (Japan) with a Sennheiser ME66/K6 shotgun microphone (Germany). Recordings were made at 192 kHz sampling rate and 32-bit float encoding. The ambient air temperature and humidity were obtained using a Huashengchang DT-83 Temp & Humidity Meter (Shenzhen, China). The Z-weighted sound pressure level (SPL) was measured approximately 0.2 m in front of the frog using an Aihua AWA5636-1 SPL meter (Hangzhou, China). For acoustic analysis, we transformed the recordings to 24-bit encoding with Adobe Audition 2023 and then analysed them with Raven Pro v.1.6.5 (K. Lisa Yang Center for Conservation Bioacoustics 2025). The “call-centred” terminology of Köhler et al. (2017) was used to describe the calls, in which the fundamental unit was defined as a “call” and the continuous units were defined as a “call group” (Qian et al. (2024): fig. S2). Terminology for acoustic measurements follows Qian et al. (2023a): call duration (ms); call interval (ms); number of calls per call group; call repetition rate (calls/s), measured by counting the total number of calls (*k*) within a call group and dividing k-1 by the duration between the onset of the first call and the onset of the last call of the call group (modified from Bee et al. (2013)); number of pulses per call; dominant frequency (Hz), measured using the function “Peak Frequency” in Raven Pro. Spectrogram measurements were taken as follows: Hann window, DFT = 512 samples, overlap = 50% and Hop Size = 256 samples. For illustration of the calls, spectrograms and oscillograms were generated using the Seewave v.2.2.0 (Sueur et al. 2008) and TuneR 1.4.2 (Ligges et al. 2013) packages, with a Hanning window size of 512 samples and 50% overlap.

## Data resources

**Figure 1.**
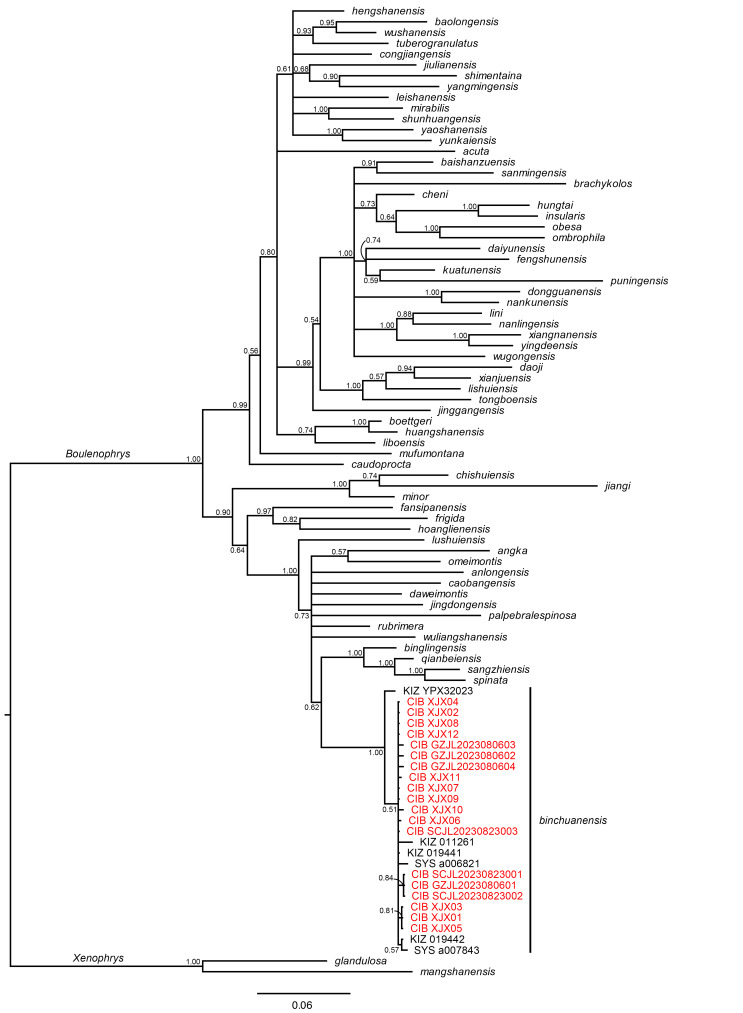
Reconstructed Bayesian topology of *Boulenophrys*, based on the 16S and CO1 genes. Numbers on nodes represent Bayesian posterior probabilities (BPP). Vouchers of new samples collected in this study are highlighted in red.

The uncorrected *p*-distance of the 16S gene within all newly-collected samples was 0.0–0.2%, and that with the previously confirmed samples of *B. binchuanensis* was 0.0–0.2%. The reconstructed topology of *Boulenophrys*, based on 16S and CO1 genes, is shown in Fig. 1. Our new samples clustered into a strongly supported clade (BPP 1.00) composed of other known *B. binchuanensis* specimens. Each node shows low genetic divergence within this clade, indicating these specimens belong to the same species.

## Taxon treatments

### Boulenophrys binchuanensis

(Ye and Fei, 1995)

78A3F546-E637-54EE-A572-87129354CBDE

#### Materials

- **Type status:** Other material. **Occurrence:** individualID: CIB GZJL2023080601–03; individualCount: 3; sex: male; lifeStage: adult; occurrenceID: 86136069-FAC1-5A13-A34D-85F5C083ADE4; **Location:** country: China; stateProvince: Sichuan; county: Jiulong; locality: Xiaojinxiang; verbatimElevation: 2540 m; verbatimCoordinates: 28.662° N, 101.925° E; **Event:** samplingProtocol: by hand; eventDate: 06-08-2023; eventRemarks: collected by Huaming Zhou; **Record Level:** basisOfRecord: PreservedSpecimen
- **Type status:** Other material. **Occurrence:** individualID: CIB SCJL20230823001–04; individualCount: 4; sex: male; lifeStage: adult; occurrenceID: 760F815E-C0A9-545C-988B-FCE695525AB1; **Location:** country: China; stateProvince: Sichuan; county: Jiulong; locality: Xiaojinxiang; verbatimElevation: 2540 m; verbatimCoordinates: 28.662° N, 101.925° E; verbatimCoordinateSystem: degrees minutes seconds; **Event:** samplingProtocol: by hand; eventDate: 23-08-2023; eventRemarks: collected by Tianyu Qian; **Record Level:** basisOfRecord: PreservedSpecimen
- **Type status:** Other material. **Occurrence:** individualID: CIB XJX01–12; individualCount: 12; lifeStage: tadpole; occurrenceID: 4B66A211-F278-5876-824C-2A19B01CDA9E; **Location:** country: China; stateProvince: Sichuan; county: Jiulong; locality: Xiaojinxiang; verbatimElevation: 2540 m; verbatimCoordinates: 28.662° N, 101.925° E; **Event:** samplingProtocol: by hand; eventDate: 23-08-2023; eventRemarks: collected by Tianyu Qian; **Record Level:** basisOfRecord: PreservedSpecimen
- **Type status:** Other material. **Occurrence:** recordNumber: CIB RC230081, 83, and 84; recordedBy: Tianyu Qian; occurrenceID: 16414D6E-5649-50FF-B2F3-5E0ADE9D535C; **Location:** country: China; stateProvince: Sichuan; county: Jiulong; locality: Xiaojinxiang; verbatimElevation: 2540 m; verbatimCoordinates: 28.662° N, 101.925° E; **Event:** eventDate: 26-08-2023; **Record Level:** type: Sound

#### Description

Morphologically, the newly -collected specimens mostly agreed with the description of Ye and Fei (1995) and the revised diagnosis proposed by Lyu et al. (2023). The following description is based on four formalin-fixed specimens (CIB SCJL20230823001–4): SVL 34.5–39.3 mm in males; small horn-like tubercles on upper eye-lid; tongue not notched posteriorly; relatively finger lengths I = II < IV < III or IV < I= II < III; fingers not webbed, lateral fringes absent or narrowly present; subarticular tubercles present on each finger; toes with rudimentary webbing, lateral fringes absent or narrowly present; subarticular tubercles distinct on all toes or indistinct on toes III and IV; metacarpal tubercles large and rounded; upper margin of tympanum concealed by supratympanic fold; dorsal skin relatively smooth, with discontinuous dorsolateral skin fold; large tubercles on flanks present. Measurements are given in Table 1.

#### Tadpoles

**Figure 2.**
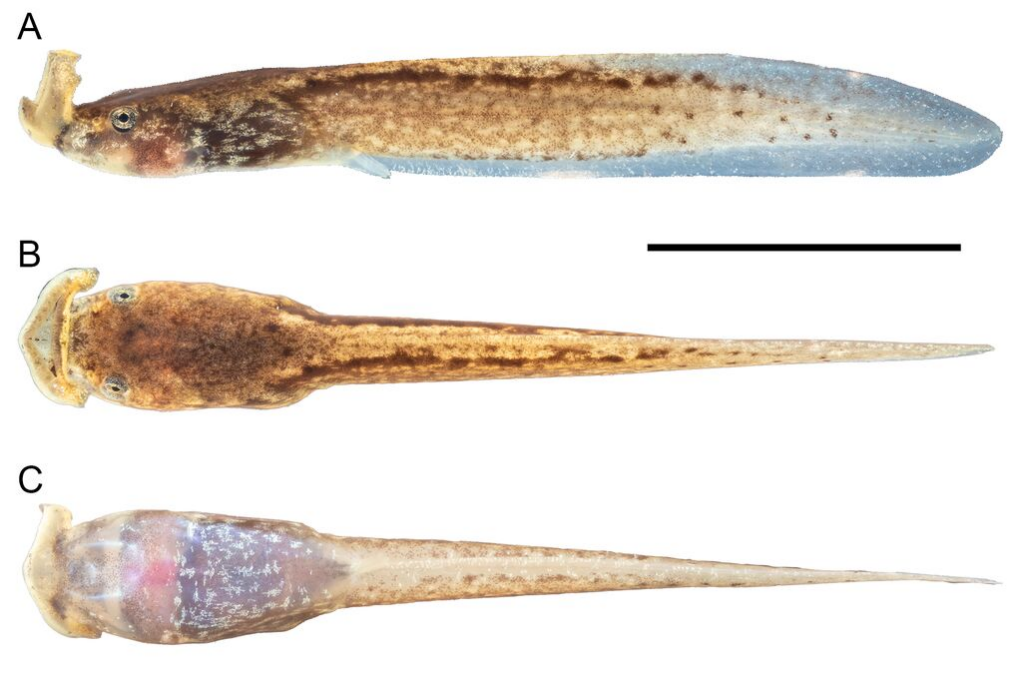
Tadpole in Stage 25 of *Boulenophrys binchuanensis* from Jiulong County. Lateral (A), dorsal (B) and ventral (C) view of CIB XJX03.

Measurements of all tadpole specimens are given in Table 2. The tadpole description is based on specimen CIB XJX03, stage 25, TTL 31.4 mm (Fig. 2). The body is elongate in lateral view, flattened and elliptical above, BW/BL 59.5%; the eyes are located dorsolaterally and not visible ventrally; the pupils are round; the nares are oval, opening laterally, closer to eye than to snout, NED/SND 66.7%; the rims of nares are slightly raised from the body wall; the spiracle is single, sinistral, low on the left body; the spiracle tube is short, free from the body wall at the tip and opens laterally, SSD/BL 56.0%; the anal tube is medial, attached to the ventral fin and opens medially, posteriorly directed; the tail muscle is strong, taller than tail fins before reaching two-thirds of the tail length, TMH/MTH 74.4%; the tail is long, accounting for 73.2% of the total length; the tail tip is rounded; the upper fin arises behind the body-tail junction, UFH/MTH 14.0%, the lower fin is connected to the trunk, LFH/MTH 18.6%; the mouth is terminal and the oral disc is funnel-like; the upper jaw sheath is wide, comb-like, exhibiting a median notch; four rows of oval submarginal papillae are visible on the upper lip and four rows of oval submarginal papillae on the lower lip; the lower jaw sheath is thin and sickle-shaped, serrated and slightly keratinised.

In life, the background colour of the body from above is yellowish and extends to the tail, scattered with brown pigments at the posterior edge of the oral disc, the inner edge of nares, the median region between eyes and above the trunk; the body is brown in lateral view, pigmented with golden speckles around the eye and on the trunk; a dark brown stripe extends alongside with the tail-upper fin connection; the tail muscle is light yellowish, scattered with brown pigments and forms several brown spots posteriorly; the body and tail are semi-transparent ventrally; brown pigments start from the lower jaw, extend medially on the throat and are widely sparse on the chest; dense whitish speckles on the abdomen and the gut coil is barely visible.

#### Advertisement calls

**Figure 3.**
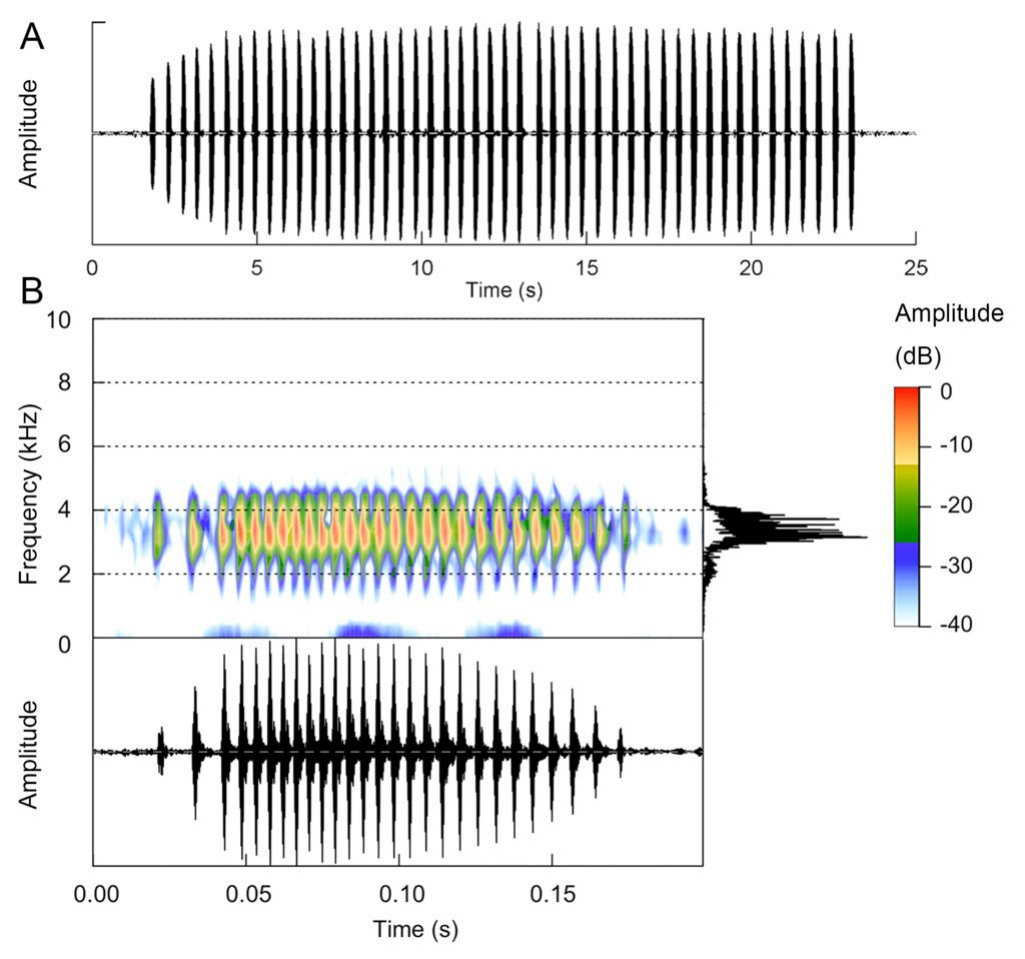
Call illustrations of *Boulenophrys binchuanensis* from Jiulong County. **A** 25 s oscillogram showing a call group produced by male frog CIB SCJL20230823004; **B** spectrogram, power spectrum and oscillogram of a call extracted from this call group.

Calls were recorded from three male frogs (see Fig. 3 for the oscillogram of a call group and a detailed illustration of a call extracted from this call group of CIB SCJL20230823004). Measurements of acoustic parameters are shown in Table 3. The calls are typical *Boulenophrys* calls with a medium number of calls per call group (averages 27, 36 and 21 from different individuals, respectively) and a medium number of pulses (averages 26, 27 and 31). The 1^st^ and 2^nd^ pulses are of relatively low amplitude with relatively larger intervals. Within a call group, the calls begin with a relatively low amplitude and increase to the peak amplitude at about the 6^th^–7^th^ calls.

#### Habitat

**Figure 4.**
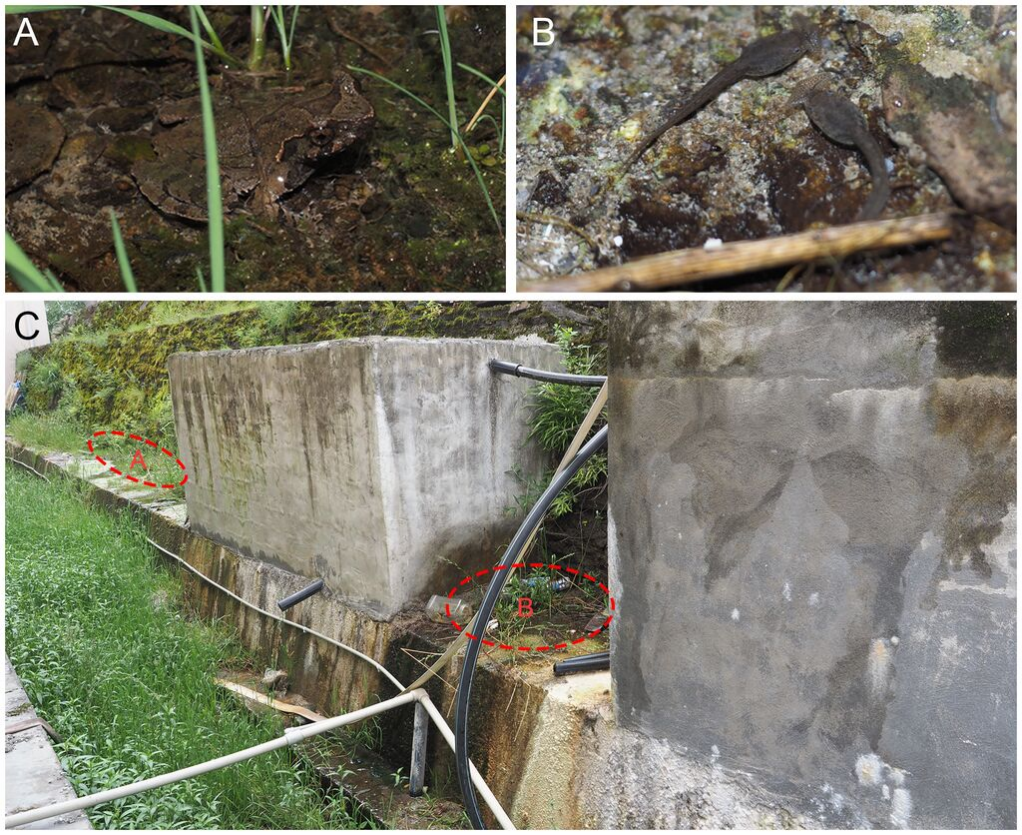
*Boulenophrys binchuanensis*. **A** Male calling frog CIB SCJL20230823002; **B** unassigned tadpoles; **C** habitat from Jiulong County.

Frogs were found in a village on the mountain top at about 2500 m in elevation. The habitat is close to an artificial reservoir; water seeps out from the wall and flows out from the water pipe (Fig. 4). Small pools form in flat areas and the ditch, which can support tadpoles. Vegetation grew in the ditch. Adult male frogs call under vegetation. We investigated two other creeks in the town; both were polluted by agricultural activities and evidence for this species was barely found.

## Discussion

Our discovery confirms the species identification of the population from Jiulong County, which was speculatively assigned, based on biogeography (Lyu et al. 2023). Consequently, the conservation status of this species should be reconsidered, as the last evaluation was based on information from only two localities (IUCN SSC Amphibian Specialist Group 2020). However, despite a larger range size, the habitat faces human disturbance from agriculture. Fortunately, this species appears to have adapted to an artificial habitat by breeding near a reservoir, which provides clean water. Further conservation efforts for this population should focus on careful management of the reservoir to maintain a wet habitat.

## Supplementary Material

XML Treatment for Boulenophrys binchuanensis

## Figures and Tables

**Table 1.** Measurements of adult specimens of *Boulenophrys binchuanensis* (in mm) from Jiulong County.

**Voucher**	**CIB SCJL20230823003**	**CIB SCJL20230823004**	**CIB SCJL20230823001**	**CIB SCJL20230823002**
Sex	Male	Male	Male	Male
SVL	38.6	34.5	36.1	35.3
HL	17.2	12.7	12.9	12.4
HW	13.4	12.2	12.7	11.5
SL	5.2	4.5	4.8	5.0
IND	4.2	3.9	4.3	4.1
IOD	3.7	3.7	3.2	3.3
ED	5.0	4.7	4.7	4.5
TD	2.8	2.5	2.2	2.3
TED	2.1	1.7	1.7	1.5
HNL	10.3	9.2	9.8	8.9
LAHL	17.5	16.5	17.5	16.6
TL	18.0	16.1	18.0	16.5
FTL	26.9	24.8	25.3	25.5
FL	17.3	16.4	17.2	16.5

**Table 2.** Measurements of *Boulenophrys binchuanensis* tadpoles (in mm) from Jiulong County.

**Voucher**	**CIB XJX01**	**CIB XJX02**	**CIB XJX03**	**CIB XJX04**	**CIB XJX05**	**CIB XJX06**	**CIB XJX07**	**CIB XJX08**	**CIB XJX09**	**CIB XJX10**	**CIB XJX11**	**CIB XJX12**
Stage	26	25	25	25	25	25	25	25	25	25	25	25
BH	4.1	3.8	4.2	3.2	4.2	3.2	3.6	4	2.9	2.6	2.8	2.7
BL	10	7.8	8.4	7	9.8	6.8	7.9	8.3	5.7	5.9	5.7	5.5
BSD	4.8	3.5	4.1	3.5	4.6	3.1	3.7	3.6	2.7	2.4	2.7	2.4
BW	4.9	4.3	5.0	3.7	5.0	3.7	4.1	4.5	3.3	2.9	3.2	3.2
ED	1.2	1.0	1.1	0.8	1.2	0.9	0.9	1.1	0.8	0.8	0.8	0.8
ESD	2.4	2.0	2.5	1.8	2.5	2.1	2.3	2.6	1.6	1.6	1.5	1.6
IND	2.8	2.2	2.2	2.0	2.6	2.0	2.2	2.3	1.6	1.4	1.8	1.8
IOD	3.3	2.9	3.2	2.6	3.5	2.8	2.9	3.3	2.4	2.2	2.2	2.2
MTH	4.2	3.7	4.3	3.4	4.3	3.3	3.7	4.0	2.7	2.4	2.5	2.8
LFH	0.9	1.0	0.8	0.8	0.9	0.6	0.6	1.0	0.5	0.4	0.4	0.6
UFH	0.8	0.9	0.6	0.7	0.6	0.5	0.6	0.8	0.5	0.5	0.4	0.4
NED	1.1	0.8	1.0	0.7	0.9	0.9	0.9	1.1	0.7	0.7	0.6	0.6
ODW	8.7	5.9	7.8	5.4	8.7	5.6	6.7	6.4	4.5	3.6	4.3	3.8
SND	1.9	1.2	1.5	1.2	1.6	1.3	1.3	1.7	1.0	1.1	1.0	1.1
SSD	4.8	4.3	4.7	3.9	5.2	4.2	4.4	4.9	3.1	3.5	3.1	3.2
TTL	32.6	26.5	31.4	18.5	32.3	24.2	27.1	32	19.1	21.6	19.2	20.8
TAL	22.6	18.7	23	11.5	22.5	17.4	19.2	23.7	13.4	15.7	13.5	15.3
TMH	3.0	2.8	3.2	2.3	3.2	2.5	3.0	3.0	2.0	1.9	1.9	2.1
TMW	2.7	2.3	2.7	2.0	2.7	2.1	2.3	2.6	1.7	1.6	1.7	1.7

**Table 3.** Measurements of male advertisement calls of *Boulenophrys binchuanensis* from Jiulong County.

**Recordings**	**CIB RC230081**	**CIB RC230083**	**CIB RC230084**
No. of calls/call groups analysed	163/6	254/7	85/4
Temperature (°C)	18.8	18.8	17.5
Humidity (%)	76.7	79.2	86.2
Sound Pressure Level (dB)	85.6	85.7	/
Call duration (ms)	149.2 ± 21.4 (31.7–263.3)	155.2 ± 7.9 (129.2–177.1)	156.8 ± 14.4 (91.2–213.9)
Call interval (ms)	304.8 ± 106.5 (63.3–1050.4)	301.0 ± 31.7 (255.2–602.1)	335.6 ± 78.0 (169.0–776.2)
Dominant frequency (Hz)	2883 ± 174 (2625–3000)	3371 ± 41 (3000–3375)	3226 ± 194 (2625–3375)
Pulse number	27 ± 4 (2–45)	26 ± 2 (19–31)	31 ± 4 (6–37)
Call rate (calls/s)	2.15 ± 0.20 (1.77–2.33)	2.20 ± 0.04 (2.15–2.25)	2.03 ± 0.04 (1.98–2.06)
Call number	27 ± 13 (10–47)	36 ± 13 (8–47)	21 ± 5 (17–29)
